# Evaluating the Impact of Motivational Enhancement Therapy on Tobacco Cessation in Schizophrenia: A Comprehensive Review

**DOI:** 10.7759/cureus.70046

**Published:** 2024-09-23

**Authors:** Asmita, Pradeep S Patil, Namita Sahu

**Affiliations:** 1 Psychiatry, Jawaharlal Nehru Medical College, Datta Meghe Institute of Higher Education and Research, Wardha, IND

**Keywords:** behavioral intervention, met, motivational enhancement therapy, schizophrenia, smoking, tobacco cessation

## Abstract

Tobacco use is markedly prevalent among individuals with schizophrenia, presenting significant challenges to their physical health and psychiatric treatment. This comprehensive review evaluates the impact of motivational enhancement therapy (MET) on tobacco cessation in this population. Schizophrenia, a chronic mental disorder characterized by symptoms such as delusions and hallucinations, is frequently accompanied by high rates of smoking, which exacerbates health risks and complicates treatment regimens. MET, a client-centered approach rooted in motivational interviewing, aims to enhance intrinsic motivation for behavior change through empathetic and non-confrontational therapeutic sessions. This review synthesizes evidence from clinical studies on MET’s effectiveness in promoting smoking cessation among individuals with schizophrenia. The review highlights the therapy’s strengths, including its adaptability and client-focused nature, which are particularly beneficial for addressing the unique challenges faced by this population. It also discusses the broader health benefits of smoking cessation, such as improved physical health and enhanced efficacy of psychiatric medications. Despite promising results, the review identifies limitations and challenges in applying MET, such as potential barriers to implementation and the need for further research. In conclusion, MET offers a valuable intervention for tobacco cessation in individuals with schizophrenia, with the potential to significantly improve health outcomes and quality of life. Future research should focus on optimizing MET strategies and exploring their broader impacts on this vulnerable population.

## Introduction and background

Schizophrenia is a chronic and debilitating mental disorder that affects about 1% of the global population [1]. Characterized by symptoms such as delusions, hallucinations, disorganized thinking, and impaired social functioning, schizophrenia significantly impacts an individual's daily life and overall quality of life. The disorder typically emerges in late adolescence to early adulthood, and individuals with schizophrenia often experience a complex interplay of symptoms and comorbidities that complicate their treatment and recovery [2]. Among these comorbidities, substance use disorders are particularly prevalent, with tobacco use being notably high in this population [2]. Tobacco use is alarmingly common among individuals with schizophrenia, with prevalence rates ranging between 60% and 90% [3]. This rate is substantially higher compared to the general population, where smoking rates are significantly lower. Several factors contribute to this increased prevalence, including the self-medication of psychiatric symptoms, the impact of antipsychotic medications, and socio-environmental influences. The high rate of tobacco use in this group underscores the need for effective interventions tailored to their unique needs [3].

The health risks associated with tobacco use are particularly severe for individuals with schizophrenia. Smoking exacerbates many physical health problems prevalent in this population, including cardiovascular diseases, respiratory issues, and a heightened risk of certain cancers [4]. Furthermore, tobacco use can interfere with the management of schizophrenia, as smoking may alter the metabolism of antipsychotic medications, potentially reducing their efficacy and leading to adverse health outcomes. Addressing tobacco use is, therefore, crucial for improving overall health and treatment outcomes in individuals with schizophrenia [4]. Smoking cessation can offer substantial benefits for individuals with schizophrenia. Quitting smoking can lead to significant improvements in physical health, reducing the risk of cardiovascular and respiratory diseases, as well as certain cancers. Additionally, smoking cessation can enhance the effectiveness of antipsychotic medications, thereby improving the management of psychiatric symptoms [5]. Beyond these health benefits, quitting smoking can improve social interactions, cognitive functioning, and overall quality of life. Thus, addressing tobacco use is a critical component of comprehensive care for individuals with schizophrenia [5].

Motivational enhancement therapy (MET) is a client-centered therapeutic approach designed to enhance an individual's motivation to change behavior. Rooted in the principles of motivational interviewing, MET focuses on resolving ambivalence and fostering intrinsic motivation for behavior change [6]. The therapy involves structured, empathetic, and non-confrontational sessions where therapists collaborate with clients to explore their reasons for change, set goals, and develop strategies for achieving them. The approach is characterized by its non-judgmental stance and emphasis on supporting the client’s motivations for change [6]. MET has been successfully applied in various contexts to address behavioral issues such as substance use disorders, obesity, and adherence to medical treatments. In the context of tobacco cessation, MET has demonstrated effectiveness in increasing motivation to quit smoking and promoting successful cessation outcomes [7]. Given its adaptable and client-centered nature, MET holds promise as a valuable intervention for individuals with schizophrenia. This population often faces unique challenges in behavior change and treatment adherence. The application of MET in this context could potentially address some of these challenges and support more successful smoking cessation efforts [7].

## Review

Mechanisms of tobacco dependence in schizophrenia

A complex interplay of biological, psychological, and social factors drives the high prevalence of tobacco smoking among individuals with schizophrenia. Understanding these mechanisms is essential for developing effective smoking cessation interventions tailored to this population [8]. Biological factors, particularly neurochemical and genetic influences, play a significant role in tobacco dependence in schizophrenia. Nicotine has been shown to alleviate certain symptoms of schizophrenia by modulating neurotransmitter systems such as dopamine, glutamate, and gamma-aminobutyric acid (GABA). This supports the "self-medication" hypothesis, which suggests that individuals with schizophrenia may smoke to compensate for neurochemical deficits associated with their condition [9]. Additionally, genetic factors likely contribute to the comorbidity of schizophrenia and nicotine dependence, with research indicating a shared genetic vulnerability that increases the risk of both disorders. Neurophysiological indices, such as those measured by electroencephalography and smooth pursuit eye movement paradigms, may be biomarkers for the underlying neuronal imbalances contributing to smoking behaviors in this population [10]. Psychological factors, including cognitive and emotional influences, also significantly affect tobacco use in individuals with schizophrenia. Nicotine may have pro-cognitive effects, improving attention, working memory, and other cognitive functions often impaired in this population. Furthermore, smoking may serve as a psychological tool for managing negative emotions, reducing stress, and coping with challenging environmental conditions [11]. Personality traits such as higher neuroticism and anxiety are frequently associated with smoking behaviors in schizophrenia, suggesting that anxiety, as a symptom of the disorder, may contribute to tobacco use. This interplay between cognitive deficits and emotional regulation highlights the complexity of smoking dependence in individuals with schizophrenia [12]. Social and environmental factors further complicate tobacco dependence among individuals with schizophrenia. Socioeconomic challenges, such as low income and unemployment, are more prevalent in this population and are associated with higher smoking rates. Additionally, many mental health facilities maintain a permissive smoking culture, which reinforces smoking behaviors through practices like providing tobacco as a reward for compliance or good behavior [13]. Institutionalization can also lead to boredom, prompting individuals with schizophrenia to use tobacco as a means of alleviating monotony and passing the time. These social dynamics underscore the importance of considering the broader environmental context when addressing tobacco dependence in this vulnerable population [3]. Key mechanisms underlying tobacco dependence in individuals with schizophrenia are summarized in Table 1.

**Table 1:** Key mechanisms underlying tobacco dependence in individuals with schizophrenia nAChRs: nicotinic acetylcholine receptors

Mechanism	Description	Impact on Tobacco Use
Nicotine's effect on dopamine release [14]	Nicotine increases dopamine levels in the brain, providing a sense of pleasure and reward	Individuals with schizophrenia may use nicotine to self-medicate, increasing dependence
Cognitive dysfunction [11]	Schizophrenia is associated with cognitive impairments such as memory, attention, and executive functioning	Tobacco use may improve attention and working memory, reinforcing the habit
Negative symptoms [2]	Schizophrenia often involves negative symptoms such as lack of motivation and social withdrawal	Nicotine may temporarily alleviate these symptoms, encouraging repeated use
Altered nicotine receptor sensitivity [15]	Schizophrenia may involve alterations in the brain’s nAChRs	This altered sensitivity could enhance nicotine’s reinforcing effects, increasing dependence
High stress and anxiety levels [16]	High levels of stress and anxiety often accompany schizophrenia	Nicotine is used as a coping mechanism to relieve stress, fostering addiction
Impaired impulse control [17]	Individuals with schizophrenia often exhibit poor impulse control and higher susceptibility to addiction	Difficulty in resisting cravings or controlling tobacco use, leads to higher dependence
Comorbid substance abuse [18]	Schizophrenia frequently co-occurs with substance use disorders, including alcohol and drugs	The presence of other addictions may heighten nicotine dependence
Social isolation and marginalization [19]	Many individuals with schizophrenia face social isolation and stigma	Smoking may become a social or coping activity, reinforcing habitual use

Motivational enhancement therapy and its application

Theoretical Framework of Motivational Enhancement Therapy

MET is a client-centered counseling approach to bolster an individual's motivation for change. Rooted in the principles of motivational interviewing, MET emphasizes several key components. One of its foundational elements is personalized feedback, where the therapist offers tailored insights based on the individual's specific behaviors and circumstances [20]. This feedback helps clients identify the discrepancies between their current behavior and personal goals, which can be pivotal in fostering a desire for change. MET also strongly emphasizes goal setting, encouraging participants to define their aspirations for change. This process cultivates a sense of ownership and commitment to their recovery journey [20]. Another critical aspect of MET is the exploration of ambivalence. Many individuals harbor mixed feelings about changing their behaviors, and MET addresses this by helping them articulate both their reasons for wanting to quit smoking and the obstacles they face in doing so [21]. MET is conducted in a supportive and non-judgmental environment and facilitates open dialogue and self-exploration, which are essential for promoting behavior change. These strategies collectively empower individuals to take an active role in their recovery and inspire them to pursue their smoking cessation goals [21].

Motivational Enhancement Therapy in Tobacco Cessation

In the context of tobacco cessation, MET targets smoking behaviors through several key mechanisms. One of its primary objectives is to increase an individual's readiness to change. By helping individuals recognize the negative consequences of smoking and the benefits of quitting, MET effectively boosts their motivation to engage in cessation efforts. Additionally, the therapy builds confidence by assisting individuals in developing concrete plans and strategies to quit smoking, thereby enhancing their self-efficacy and belief in their ability to succeed [7]. MET also addresses the specific barriers that individuals encounter when trying to quit smoking. Through open discussions, the therapy enables individuals to strategize around common challenges such as cravings, withdrawal symptoms, and social pressures, providing them with practical tools to overcome these obstacles [22]. Moreover, MET facilitates behavioral change by utilizing motivational interviewing techniques that help individuals identify and implement strategies that support their cessation efforts, such as avoiding triggers and developing coping mechanisms. MET offers a comprehensive framework for those seeking to quit smoking by enhancing motivation, building confidence, and addressing the barriers to cessation [22].

Adaptations of motivational enhancement therapy for schizophrenia

When applying MET to individuals with schizophrenia, several modifications and considerations are necessary to ensure its effectiveness. Understanding the unique psychosocial challenges faced by this population is crucial, as individuals with schizophrenia may encounter barriers such as social isolation, cognitive impairments, and higher levels of nicotine dependence. Therefore, MET must be specifically adapted to address these challenges effectively [23]. Involving family members or caregivers in the MET process can provide additional support and encouragement, which is particularly important for individuals with schizophrenia who may struggle with motivation and social interactions. Engaging these supportive figures helps create a more robust support system, enhancing the individual's commitment to the cessation process. Flexibility in the delivery of MET is also essential, as individuals in this population may exhibit varying levels of cognitive function and motivation. This flexibility allows the therapist to adjust the approach based on the individual’s current mental state and readiness to engage in the process, ensuring that the therapy remains accessible and effective [24]. Additionally, integrating MET with pharmacological treatments and other psychosocial interventions, such as cognitive-behavioral therapy (CBT), can significantly enhance its effectiveness. These combined approaches address both the psychological and physiological aspects of nicotine dependence, making them particularly suitable for individuals with schizophrenia. By targeting multiple facets of tobacco dependence, this integrative strategy can lead to more comprehensive and lasting outcomes [25]. While MET is a promising approach to supporting tobacco cessation among individuals with schizophrenia, tailoring the therapy to meet their unique needs and challenges is vital for optimizing its application and effectiveness [25]. Key components of MET and their application in tobacco cessation are outlined in Table 2.

**Table 2:** Key components of MET and their application in tobacco cessation SMART: specific, measurable, achievable, relevant, and time-bound; MET: motivational enhancement therapy

Component of MET	Description	Application in Tobacco Cessation
Assessment and feedback [26]	A structured evaluation of the patient’s tobacco use, including motivations, barriers, and readiness to change	Use tools like motivational interviews and self-assessment questionnaires to understand tobacco dependence levels
Developing discrepancy [27]	Helping the patient recognize the gap between their current behavior (tobacco use) and future goals (cessation)	Discuss how continued smoking contradicts the patient's long-term health and personal life goals
Expressing empathy [28]	Building a non-judgmental and understanding relationship with the patient, creating a supportive environment	Use empathetic communication to encourage openness and self-reflection regarding tobacco habits
Enhancing motivation [29]	Encouraging the patient to explore their reasons for quitting and reinforcing their motivation to change	Focus on the benefits of quitting smoking, such as improved health, finances, and social relationships
Rolling with resistance [30]	Avoiding confrontation by accepting and acknowledging the patient’s ambivalence toward quitting	Respect the patient’s pace and reasons for hesitation, offering gentle guidance rather than pushing
Supporting self-efficacy [31]	Empowering the patient to believe in their ability to quit smoking and sustain abstinence	Highlight past successes in quitting or managing difficult tasks to boost confidence in quitting tobacco
Goal setting and action plan [32]	Creating SMART goals for smoking cessation	Work with the patient to set small, realistic steps toward cessation, such as reducing the number of cigarettes
Ongoing monitoring and adaptation [33]	Continuously assessing progress and adapting strategies to suit the patient’s evolving needs and challenges	Regular follow-ups, adjusting goals or techniques based on progress, and addressing any relapses

Review of clinical studies and evidence

Several clinical studies have explored the effectiveness of MET for smoking cessation among individuals with schizophrenia. One notable study focused on a tailored smoking cessation program specifically designed for this population, reporting significant short-term abstinence rates. These findings suggest that structured interventions like MET can effectively facilitate quitting among individuals who face unique mental health challenges. Additionally, research has shown that MET can significantly increase motivation and self-efficacy in smokers with schizophrenia, helping them navigate the complexities of quitting. Importantly, studies indicate that combining MET with pharmacotherapy, such as nicotine replacement therapy (NRT) or bupropion, may enhance cessation outcomes by addressing both the psychological and physiological aspects of nicotine dependence [34]. The effectiveness of MET in promoting smoking cessation among individuals with schizophrenia has been well-documented. For instance, one study found that 42% of participants who completed a seven-week MET group program successfully quit smoking, with 12% remaining abstinent at a six-month follow-up [35]. While these abstinence rates are lower than those typically observed in the general population, they still represent a significant improvement, especially when MET is combined with pharmacotherapy and tailored to the individual needs of participants. The enhancement of motivation and commitment to quitting that MET fosters is crucial for individuals with schizophrenia, who often experience heightened anxiety and stress that can complicate cessation efforts [35]. Despite the promising results associated with MET, there are notable limitations and challenges in applying this approach to individuals with schizophrenia. Many studies have small sample sizes, which can limit the generalizability of the findings, and long-term follow-up data is often lacking. Additionally, individuals with schizophrenia may face heightened anxiety, stress, and cognitive deficits that can complicate their ability to engage in and benefit from MET [36]. Furthermore, the normalization of smoking within psychiatric settings and the lack of structured support for cessation can hinder the implementation and success of MET programs. To address these challenges, it is essential to create supportive environments that encourage cessation, provide continuous follow-up, and tailor interventions to the specific needs and barriers faced by individuals with schizophrenia. By doing so, healthcare providers can improve the likelihood of successful smoking cessation and enhance the overall well-being of this vulnerable population [37].

Integration of motivational enhancement therapy into clinical practice

Clinicians must prioritize comprehensive training and education on MET principles and techniques to effectively integrate MET into clinical practice. Such training should equip healthcare providers with the necessary skills to engage patients meaningfully, using motivational interviewing strategies that foster a supportive therapeutic environment [6]. A patient-centered approach is essential, with clinicians focusing on individual patient needs, preferences, and readiness to change. By actively listening and demonstrating empathy, clinicians can build rapport and trust, which is crucial for encouraging patients to explore their motivations for quitting smoking [38]. Setting clear, achievable goals in collaboration with patients can significantly enhance their motivation and commitment to change. Clinicians should assist patients in identifying personal reasons for quitting and the benefits they hope to gain from cessation [39]. Regular follow-up appointments are also vital, as they provide ongoing support and motivation. These check-ins allow clinicians to monitor progress, celebrate successes, and adjust treatment plans. Additionally, clinicians should be aware of and utilize available resources, such as smoking cessation programs and pharmacotherapy options, to offer comprehensive support to their patients [39]. Implementing MET in clinical practice often encounters barriers that must be addressed to ensure successful outcomes. One significant barrier is the stigma surrounding mental illness and smoking [40]. To combat this, it is crucial to educate both staff and patients about the importance of smoking cessation in mental health. Promoting a supportive environment can encourage individuals to seek help and engage in cessation efforts. Enhancing accessibility to MET and smoking cessation resources within clinical settings is another strategy to encourage participation, whether through integrating these services into routine care or offering them in various formats, such as in-person sessions or telehealth options [40]. Professional training programs are pivotal in increasing healthcare providers' likelihood of offering MET and smoking cessation assistance [41]. These training sessions should focus on the specific needs of patients with mental illness, equipping providers with the tools to address their unique challenges. Furthermore, establishing supportive policies that promote smoking cessation as a standard part of care for individuals with mental health conditions can help institutionalize these practices, making them an integral component of mental health treatment [41]. Looking ahead, several areas warrant further research and development to optimize the integration of MET into clinical practice. One critical area is the continued evaluation of the long-term effectiveness of MET in diverse clinical settings, particularly among populations with severe mental illness. Research should focus on MET adaptations and outcomes to determine the most effective strategies for promoting smoking cessation [42]. Additionally, exploring the use of technology, such as mobile health applications and telehealth services, to deliver MET and support smoking cessation could enhance accessibility and engagement. Understanding the specific barriers faced by patients with mental illness in accessing and engaging with MET and smoking cessation programs is also essential. Identifying these barriers can inform tailored interventions that address the unique challenges of this population [43]. Comparative studies between MET and other behavioral interventions can provide valuable insights into the most effective strategies for smoking cessation in individuals with schizophrenia and other mental health disorders. Lastly, research should focus on developing policies supporting MET implementation in clinical practice, ensuring that smoking cessation is prioritized in mental health care settings [44]. By addressing these areas, the integration of MET can be optimized, ultimately improving smoking cessation outcomes for individuals facing mental health challenges [45]. A framework for integrating MET into clinical practice for tobacco cessation in schizophrenia is shown in Table 3.

**Table 3:** Framework for integrating MET into clinical practice for tobacco cessation in schizophrenia NRT: nicotine replacement therapy; MET: motivational enhancement therapy

Step/phase	Description	Implementation strategies	Expected outcomes
Initial assessment and engagement [46]	Evaluate tobacco use, motivation level, and readiness for change in patients with schizophrenia	Use standardized tools such as the Fagerström test for nicotine dependence and motivational interviewing techniques	Build rapport and gain insight into the patient's tobacco use patterns and motivation level
Setting personalized goals [47]	Collaborate with the patient to set achievable and personalized tobacco cessation goals	Tailor goals are based on the patient’s readiness to quit, cognitive capacity, and individual preferences	Increased patient engagement and commitment to the quitting process
Tailoring MET Sessions [48]	Adapt MET sessions to address schizophrenia-specific challenges such as cognitive impairments and comorbidities	Use simplified language, visual aids, and repetition. Focus on schizophrenia-related smoking triggers	Enhanced understanding and retention of MET strategies, fostering behavior change
Integration with pharmacotherapy [49]	Combine MET with NRT or other cessation medications like bupropion or varenicline	Regularly assess the effectiveness of the pharmacotherapy and make adjustments as needed	Improved smoking cessation outcomes through combined behavioral and pharmacological approaches
Monitoring and relapse prevention [50]	Continuous monitoring of progress and addressing potential relapses	Schedule regular follow-up visits, use telehealth when necessary, and maintain MET reinforcement sessions	Reduced relapse rates and sustained abstinence from smoking
Multidisciplinary collaboration [51]	Work closely with psychiatrists, psychologists, and other mental health professionals	Ensure communication between healthcare providers for coordinated care	Holistic patient care ensures both mental health and tobacco cessation needs are addressed
Addressing comorbid substance use [52]	Identify and manage co-occurring substance use disorders in patients with schizophrenia	Integrate MET strategies with broader substance use treatment plans	Improved overall addiction management and support for quitting tobacco
Family and social support involvement [53]	Engage family members or caregivers in the MET process to support the patient’s cessation efforts	Provide education to families about schizophrenia and tobacco dependence and involve them in MET sessions when appropriate.	Increased social support and accountability, fostering greater success in cessation.

## Conclusions

In conclusion, the integration of MET into tobacco cessation strategies for individuals with schizophrenia represents a promising approach to addressing a critical health challenge within this population. Given the high prevalence of tobacco use and its detrimental impact on both physical health and psychiatric treatment, effective cessation interventions are essential. MET's client-centered, empathetic approach aligns well with the needs of individuals with schizophrenia, offering a tailored method to enhance motivation and facilitate behavior change. The evidence supporting MET's effectiveness in various contexts, combined with its potential benefits for managing schizophrenia, underscores the importance of incorporating this therapeutic approach into comprehensive care plans. Future research and clinical practice should optimize MET strategies for this unique population, address implementation challenges, and further explore its impact on health outcomes and quality of life. By prioritizing tobacco cessation through evidence-based interventions like MET, we can improve overall well-being and treatment outcomes for individuals with schizophrenia.
